# A Case for Diagnosis

**Published:** 1908-01-25

**Authors:** 


### A CASE FOR DIAGNOSIS.

A young man, aged 21, was found in bed comatose one morning. The history was that he had had typical chorea three times as a youth, the last attack occurring two years before the present trouble. He had worked as a clerk in an office. His habits were very intemperate as regards alcohol, but there was no history of syphilis. Six months ago he had an illness of some kind, during which he was dazed and unable to speak properly, the partial aphasia lasting a week; it was not thought much of at the time, being attributed to the effects of drink. He recovered from this attack, but continued to drink heavily at intervals. After one heavy bout of intoxication he was put to bed, sleeping soundly, and next morning it was found impossible to awaken him. He was comatose, and he remained so throughout that day; on the next he had partly recovered consciousness, but he was unable to speak, though he made attempts to do so. There was no paralysis of arm or leg, nor of any cranial nerve; the reflexes were normal; he could understand things said to him, and would shakily and slowly write his name when asked to do so; he knew the uses of things shown to him as far as could be judged, though he was in a generally dazed and stupefied condition, with very slow cerebration. He made unintelligible noises now and then, but never spoke actual words. For a month he remained in much the same condition, getting neither better nor worse, and it was suggested that he was suffering from alcoholic dementia from general degeneration of the cerebral cells. The physical signs in his lungs were at that time natural; there was no cardiac bruit, and the heart sounds were normal; no pain was complained of anywhere; there was no vomiting, no optic neuritis, no discharge from the ears; neither liver nor spleen was palpable; the urine contained neither albumen nor sugar, and a fair quantity was passed daily. The pulse rate was slightly above normal, the respiration rate was natural, and the temperature was natural except for an occasional rise to just over 99° F.

A month after the attack began the stupor increased, relatives were not recognised, and urine and fæces were passed into the bed; nasal feeding had to be resorted to; the condition was not one of absolute coma, but one of profound stupor. There was never any convulsive seizure. Shortly after this the temperature, pulse rate, and respiration rate all rose; the percussion note over the upper lobe of the right lung became very impaired, and bronchial breathing with crackling râles developed in this region next day; two days later there were similar impairment of note, bronchial breathing, and crackling râles in the left axilla; the abnormal physical signs increased in both lungs; there was no sputum; he lived for a fortnight after the lung trouble was first detected, the cerebral condition remaining much the same until the end. There was no bruit to be heard in the heart at any time, and the urine, though it became concentrated, never contained any albumen.

The diagnosis was not cleared up until the autopsy was made next day.

### SOLUTION.

There had been endocarditis in connection with the attacks of chorea; fungating endocarditis had supervened, leading to an older embolism of the left Broca's area, and a more recent one on the corresponding area of the right side. The attack of aphasia six months before death was clearly due to the former embolism, the second being the cause of the final attack with its aphasia. The lungs were the seat of a terminal lobar and broncho-pneumonia, which were the immediate cause of death.

The following is a more detailed account of what was found at the autopsy :—

The patient was a very thin, dark-haired, under-sized youth. There were no petechiæ or other skin lesion. Rigor mortis was well marked, hypostasis moderate.

The *brain* weighed 1,133 grams. There was an area of cortical softening on each cerebral hemisphere, each patch about half an inch deep, bounded all round by firm brain substance. The softening on the left side was confined to Broca's area, and it was obviously of some months older date than the other. The patch on the right side affected not only the pars triangularis, but also a long strip of the cortex behind this, parallel with and above the Sylvian fissure, but not extending far enough up on to the hemisphere to involve the arm area. Each patch of softening was clearly due to blocking of branches of the middle cerebral arteries from embolism. The rest of the brain, its meninges, ventricles, and vessels, were all natural.

The *pleuræ* were widely adherent by old fibrous tissue to chest wall, diaphragm, and pericardium, and in addition there was acute recent pleurisy all over the left lower lobe, and over the greater part of all the lobes on the right side. There was no free fluid nor pus in either pleural cavity.

The *lungs* were voluminous and heavy; the only breathing-lung was in the left upper lobe, and in small portions of the two lower lobes; all the rest was consolidated by a mixed condition of broncho- and lobar-pneumonia. There was nowhere in the body any sign of tubercle.

The *heart* had no pericarditis, but the muscle of both right and left ventricles contained numerous miliary abscesses in its substance. There was no particular dilatation or hypertrophy. The tricuspid, pulmonary, and aortic valves were healthy. The mitral valve was neither incompetent nor stenosed, but it was much diseased; there was some thickening of its edge, due, no doubt, to endocarditis at the time of one or other of the patient's attacks of chorea; in addition to this there were three large irregular masses of fungating fibrin arising from three separate foci on the valve, and spreading up towards the auricle; there were no bead-like granulations, but only these fungating masses, the largest of which was as big as a small bean, but quite irregular. They were firm and pale, and it seemed quite probable that they had been growing for six months or more, so that they were the source of both cerebral emboli. There was no antemortem thrombosis within the heart.

The *liver* was of good shape and consistence, neither cirrhotic, fatty, nor nutmegged.

The *stomach and intestines* were natural.

The *spleen* was slightly enlarged, pale, and fairly firm; it contained no recent infarct, but there was a deep depressed scar due to the organisation of an infarct of long ago.

The *kidneys* showed none of the petechiæ usually seen in cases of fungating endocarditis; there were several deep scars due to the complete organisation of former infarcts, and there were two other depressed, but not quite organised infarcts which probably dated from only some months back. There was no recent infarction.

The other viscera showed no abnormalities.

Bacteriological examinations of the organs showed that streptococcus longus was present, as well as bacillus coli-communis; it is, therefore, probable that the fungating endocarditis was of streptococcal origin, superimposed upon a valve slightly damaged by rheumatic inflammation during former chorea.

